# Oncology nurses’ experiences of providing emotional support for cancer patients: a qualitative study

**DOI:** 10.1186/s12912-024-01718-1

**Published:** 2024-01-20

**Authors:** Xiao-Chen Lyu, Hai-Jiao Jiang, Li-Hung Lee, Cheng-I. Yang, Xiang-Yun Sun

**Affiliations:** 1 Department of Nursing, First Affiliated Hospital of Wannan Medical College, Anhui, China; 2 Intensive Care Unit, First Affiliated Hospital of Wannan Medical College, Anhui, China; 3 Department of Nursing, Hungkuang University, No. 1018, Sec. 6, Taiwan Boulevard, Shalu District, Taichung City, 433304 Taiwan; 4 Department of Oncology, First Affiliated Hospital of Wannan Medical College, Anhui, China

**Keywords:** Cancer patients, China, Emotional support, Oncology nurses, Qualitative study

## Abstract

**Background:**

A high percentage of cancer patients may experience emotional distress. Oncology nurses are expected to play an important role in recognizing emotional distress and planning and delivering care that meets the individual needs of each patient. However, few studies have focused on the experiences of clinical nurses in such cases. This study adopted a qualitative research method to gain an in-depth understanding of the experience of nursing staff in caring for cancer patients with emotional distress.

**Methods:**

A qualitative descriptive design and semi-structured interviews were used in this study. Twenty-one oncology nurses were interviewed, and the qualitative content analysis suggested by Graneheim & Lundman (2004) was used to interpret the data.

**Results:**

Six themes were identified, as follows: (1) dictating the abnormality of emotion, (2) soothing and comforting patients, (3) a lack of psychology knowledge and communication skills, (4) negative impacts of a lack of time, (5) managing emotional labor, and (6) reflecting on the experiences.

**Conclusion:**

Hospital administrators should arrange pre-employment education and training as well as on-the-job education to help nurses in caring for cancer patients with emotional distress. They should also focus attention on the personal emotional states of nursing staff in a timely manner and provide psychological support and emotional counseling as necessary.

## Background

Cancer is the second leading cause of death globally. The global cancer burden is projected by the World Health Organization (WHO) to rise from 19.2 million in 2020 to 21.6 million by 2030, and 9.6 million deaths were recorded in 2018 alone [1]. Advances in cancer treatment have increased survival rates [2]. Worldwide, the total number of people who are alive 5 years after a cancer diagnosis (5-year prevalence) is estimated to be 43.8 million [3]. When a person has cancer, emotional distress can affect the person’s finances, family, sexuality, spirituality, and many other aspects of life [4]. It is quite clear then that the needs of patients with cancer are not limited only to the physical aspects related to the disease and its treatment; rather, they include a wide range of emotional, inter-personal and social implications, the consequences of which should be constantly monitored across the illness trajectory for both patients and family members [5]. Furthermore, patients with cancer experience varying levels of emotional distress throughout the disease trajectory associated with the diagnosis of cancer, as well as the effects of the disease and treatments.

Emotional distress is a general term used to describe negative feelings or emotions that affect one’s level of functioning and interfere with activities of daily living [6]. The National Comprehensive Cancer Network (NCCN) defines emotional distress as a multifactorial unpleasant emotional experience of a psychological, social, and/or spiritual nature that may interfere with the ability to cope effectively with cancer, its physical symptoms, and its treatment [7]. Emotional distress may also compromise the quality of life, and it has been recognized as the sixth vital sign (in addition to pulse, respiration, blood pressure, temperature and pain) to be frequently and regularly monitored in the cancer care course [7]. Failure to acknowledge such distress early on may greatly increase the severity of patients’ cancer symptoms and negatively impact their well-being and quality of life (QOL), and the oncological process itself [8].

Emotional Distress is common among cancer patients. Cancer patients across the trajectory of illness (i.e., newly diagnosed to years post treatment) with different cancer diagnoses reported that 23–38% experience clinically significant distress [9]. Similarly, studies show that more than 43% of patients with cancer suffer significant emotional distress [10]. More than 12 million cancer survivors are living in the United States; among them, 20%–40% experienced high levels of distress during their treatment [11]. The relevant statistics are even higher, as the patient's psychosocial needs may not be perceived by either the patient or the professional during the course of the patient's cancer development [3]. Additionally, studies suggest that distress is present among different cancer types, at different points in the disease trajectory, and across different countries, making it a pertinent issue for all cancer survivors [9].

Nurses spend more time with patients than any other member of the healthcare team. The scope and standards of oncology nursing practice prepare nurses to collect in-depth data regarding the “physical, psychosocial, social, spiritual, and cultural health status of patients [12]; therefore, oncology nurses are expected to be able to identify emotional distress and to plan and provide nursing care that meets each patient’s individual needs [13]. From some oncology nurses' perspectives, providing cancer patients with psychological support are including caring compassionately with emotional support, having a friendly relationship with the patient and having communicative behavior facing patient needs [14]. Unfortunately, previous research found that most healthcare professionals (HCPs), including nurses, find it difficulty, and frequently fail to recognize such symptoms among patients [8]. Barriers to identifying and caring for patients’ distress may concerns to the factors of patients concealing distress, nurses’ lack of training, and time constraints [15]. Additionally, oncology nurses may lack of knowledge and skill and worry about how to care with patients’ distress of anxiety and frustration [16]. They are in need of support and necessary assistance to help them change their own perceptions on cancer, to become emotionally stronger, and to acquire necessary knowledge and skills to provide patients with emotional care [17].

Although it is common knowledge that a high proportion of cancer patients may suffer from emotional distress, there is also a consensus that early detection of patients' psychological care needs is required for early intervention or assistance. However, in the past research literature, few relevant studies have focused on how nurses in cancer wards actually provide psychological support to patients. Additionally, most of them were too old and conducted in Western countries, and a lack of the understanding the phenomenon in the context of China. Therefore, this study intends to adopt a qualitative research method to gain an in-depth understanding of the experience of nursing staff in caring for cancer patients with emotional distress, and then to provide suggestions for the quality of clinical care and related education policies.

## Method

### Research design

A qualitative descriptive design [18] with semi-structured interviews was used in this study. A qualitative descriptive design is frequently used in nursing and healthcare research, as such an approach can provide broad insight into particular phenomena and can be used in a variety of ways [18]. It is especially helpful in providing straightforward descriptions of experiences and perceptions, particularly in areas where little is known about the topic under investigation [19]. This design allowed us to describe the nurses’ experiences of and perspectives on caring for cancer patients with emotional distress.

### Sampling and participants

In this study, the participants were recruited from the oncology wards of two different hospitals in the Anhui Province of China. This study was reviewed and approved by an Ethics Committee of a medical college. After obtaining ethical approval, the first author contacted the managers of the hospitals to ask for a list of potential participants who met three inclusion criteria: (a) full-time, registered nurse working in the oncology wards of the hospital, (b) at least one year of experience working with patients diagnosed with cancer, and (c) willingness to participate in this study. Twenty-five nurses who fulfilled the basic recruiting requirements and expressed interest in participating in research were recommended by the managers of the hospitals. All 25 nurses were invited by the first author. Four later decided not to participate because of time conflicts or for personal reasons, leaving 21 nurses who were willing to participate in this study. The four non-participating nurses were not significantly different from the other participants in age, gender, educational level and nursing work experience.

### Data collection

Prior to the interviews, the participants were informed of the purpose and procedure of the study, their rights in participating, and the recording of interviews with a voice recorder. After written consent was obtained, the interviews were conducted in a small conference room at the hospital where the participants could feel comfortable or by online interview, according to the wishes of the participants.

All the interviews were performed individually by the first author using a semi-structured interview guide (Table 1). Each participant was interviewed once, and each interview lasted about 30–60 min. We asked participants about their experiences, perspectives, and major concerns related to caring for cancer patients with mental distress. The interviews began with an open question, such as “What are your experiences and thoughts regarding caring for cancer patients in the clinic?” Then additional interview questions were asked to further guide the participants to share more of their thoughts until the interviewer fully understood their experiences or the context of the events they described.

**Table 1:** Questions in the semi-structured interview guide

Interview questions
1. What are your experiences and thoughts regarding caring for cancer patients in the clinic?
2. During the nursing process, what do you think are the more common types of emotional distress of cancer patients?
3. How do you usually perceive or find out that cancer patients have emotional distress?
4. If you found that a patient was having emotional distress, how would you interact with the patient, or would you like to do something for him or her?
5. Can you describe a cancer patient with emotional distress that you have taken care of and have a deep impression of, and how you interacted with him or her?

After completing each interview and before proceeding with the next interview, the first author completed the transcription within 2 or 3 days, after which all authors read the transcript through and discussed and commented on the potential key themes and issues in the transcripts of the participants’ experiences. The concept of saturation has attained widespread acceptance as a methodological principle in qualitative research [20]. After the interview of the 19th participant, we found that no new information had been gained from the interview. We interviewed 2 more participants, and no new information related to the research was obtained. Perceiving that we had collected sufficient data and achieved data saturation, we decided to discontinue the interviews [21].

### Data analysis

The 21 interviews were transcribed verbatim by the first author and analyzed using the qualitative content analysis suggested by Graneheim & Lundman [22]. Initially, each entire transcript was read and reread by the authors to obtain an overall understanding of the text and to familiarize the authors with the participants’ experiences of and concerns about caring for cancer patients with emotional distress. Each author used highlighters to highlight segments of the texts. Each highlighted segment was assigned a name as the initial code to describe the idea or feeling expressed in that part of the text. We then shared and discussed the highlighted segments and codes which we perceived as important and relevant to the study issues until consensus was reached. The relationships between codes and themes were carefully considered and examined during the process of analysis. In the end, six themes were identified. An example of coding is showed in Table 2.

**Table 2:** An example of codes, subthemes and theme from content analysis of narratives about theme one

	Codes	Subthemes	Theme one
Because I work in the Medical Ward mainly involves intravenous lines and infusion treatments, I don’t have much time to chat with patients. However, every time during the infusion, I observe whether his/her mood is good or not.	Observing the patient's condition	Assessing patient emotions through daily interaction	Dictating the abnormality of emotion
First, chat with him slowly. Start with the simplest questions. Ask him how his food was today and whether he went out for a walk. See if he is willing to talk to you.	Assessing through interaction		
First of all, I observe a patient’s expression. Everyone's emotions are different, and their expressions must be different. Also, observe whether the patient is willing to communicate with you. We usually talk to the patient, and some patients are unwilling to communicate.	Unwilling to communicate	The warning signs of emotional abnormality	
We will notice some patients lying on the bed or sitting in a chair alone, and looking dull. At this time, we feel that her mood may be a little low.	Being alone		
There are also some patients who are more obvious. She may secretly wipe her tears. This is something we can observe.	Crying		
Basically, we all know patients who are repeatedly hospitalized. We know these patients’ personalities and their family situations. When you feel that a patient’s reaction is not the same as before, you may pay more attention to them, so that it is easy to find.	Patient’s reaction change		

### Trustworthiness

The concept of trustworthiness was kept in mind throughout the whole process of the study, including the data collection, analysis and presentation [23]. First, all authors had expertise in oncology, psychiatric nursing or qualitative research, which allowed a fuller understanding of the participants’ experiences. Second, the study findings were derived from several different sources of data. The study included participants from different wards and hospitals and with different working experiences to increase the possibility of understanding oncology nurses’ experiences from a variety of perspectives [22]. Third, all the interviews were conducted by the first author, and during the process of data analysis, the authors met regularly to discuss and check the coding, sorting and naming for verification, as suggested by Coleman [23]. Fourth, we extracted participants’ experiences as thick descriptions of the study phenomena for each theme. Additionally, we used the 10 questions and their suggested belonging considerations of the CASP qualitative checklist to examine the whole paper so as to enhance the trustworthiness and quality of the paper [24].

## Findings

In all, 21 oncology nurses participated in this study. All of them were female and had bachelor’s degrees. Their age ranged from 25 to 40 years old (mean = 33.0). Their total nursing work experience ranged from 2 to 18 years (mean = 10.3), while their experience as oncology nurses ranged from 1 to 17 years (mean = 9.1). Table 3 shows the detailed demographic characteristics of the participants. Additionally, none of them had received any formal psychological or mental healthcare training regarding the care of cancer patients. Data analysis revealed that the participants’ experiences of caring for cancer patients with mental distress were captured by six themes and 13 sub-themes, as listed in Table 4.

**Table 3:** Participant characteristics (*N* = 21)

	Numbers	Percentages
Age (year)	Range = 25–40	Mean = 33.0
20–25	2	9.5%
26–30	4	19.0%
31–35	9	42.9%
36–40	6	28.6%
Hospital		
A in Anhui Province	15	71.4%
B in Anhui Province	6	28.6%
Nursing work experience (Year)	Range = 2–18	Mean = 10.3
1–5 years	5	23.8%
6–10 years	4	19.0%
11–15 years	10	47.7%
16–20 years	2	9.5%
Experience in the oncology wards (Year)	Range = 1–17	Mean = 9.1
1–5 years	3	14.3%
6–10 years	5	23.8%
11–15 years	8	38.1%
16–20 years	5	23.8%

**Table 4:** Themes and subthemes

Themes	Sub-themes
Dictating the abnormality of emotion	✧ *Assessing patient emotions through daily interaction* ✧ *The warning signs of emotional abnormality*
Soothing and comforting patients	✧ *Back on track with treatment* ✧ *The importance of trust in the relationship* ✧ *The help of peer support*
A lack of psychology knowledge and communication skills	✧ *Not my expertise* ✧ *A lack of communication skills*
Negative impacts of a lack of time	✧ *It takes time* ✧ *Limited time available*
Managing emotional labor	✧ *Digesting the emotions of an angry patient* ✧ *Compassion and powerlessness*
Reflecting on the experiences	✧ *Accomplishment in doing good things* ✧ *Cherish personal and family health*

### Theme 1: Dictating the abnormality of emotion

#### Assessing patient emotions through daily interaction

According to the participants’ experiences, most of the patients they encountered in the oncology wards were repeatedly in and out of the hospital for treatments such as chemotherapy and radiotherapy. Each hospital stay was not long. Although all participants believed that providing patients with so-called "psychological care" during their hospitalization was one of the important nursing tasks in caring for cancer patients, they all admitted that, based on their busy working conditions, they usually could not spend a great deal of time to carefully assess the patient's emotional problems. They tended to observe or interact with patients incidentally, through daily nursing care, and briefly assess whether the patient was emotionally distressed.

Like when I'm on an IV, or when I'm taking care of other patients in the next bed. If I hear a patient talking to others, and I feel that his emotions or tone of speech are not quite right, I will talk to him more. (Participant 5).

In fact, we are very busy with clinical work. In order to save time, I will spread my psychological care throughout my daily nursing work for patients. (Participant 19)

#### The warning signs of emotional abnormality

Most of the participants tended to focus on the completion of their various treatment tasks. A few participants also said that they would not take the initiative to talk to patients about their conditions, or ask whether they were in a good mood, because they were worried that this would irritate the patients. Only when the so-called emotional abnormality occurred in a patient would they pay more attention to the patient's emotional distress. There are many clinical signs that indicate a state of emotional abnormality, such as not speaking, being unwilling to answer questions, or appearing in a different emotional state than usual, or obviously displaying anxiety, anger, or even suicidal tendencies.

First of all, I observe a patient’s expression. Everyone's emotions are different, and their expressions must be different. Also observe whether the patient is willing to communicate with you. We usually talk to the patient, and some patients are unwilling to communicate. (Participant 7).

Depressed patients generally speak less and may be prone to depression. Anxious patients generally have more questions. For example, if you give them a stomach medicine, they will confirm it repeatedly and ask many times. Angry patients typically take out their emotions on the nursing staff. (Participant 16).

Basically, we all know patients who are repeatedly hospitalized. We know these patients’ personalities and their family situations. When you feel that a patient’s reaction is not the same as before, you may pay more attention to them, so that it is easy to find. (Participant 6).

### Theme 2: Soothing and comforting patients

#### Back on track with treatment

All the participants expressed understanding that the patient's emotions will inevitably be affected by cancer and the process of receiving treatment. However, they still expressed hopes that patients could face the disease optimistically and receive treatment. They perceived that, in this situation, it is not only the best state for patients to resist cancer but also allows the daily care work of nurses to be completed smoothly. Patients being emotionally stable and willing to cooperate with the treatment was regarded as the best interaction scenario**.** In contrast, emotional distress in patients, which is believed to negatively affect patients' attitudes and behaviors related to receiving treatment, hinders the normal operation of nursing interventions and should be eliminated so as to help patients get back on track with their treatment.

Many patients are not supported by their families. Their family members are unwilling to take care of them, and some are unwilling to pay for medical expenses. At this time, we have to comfort them in various ways, so that their emotions can be stabilized a little, so that they can receive treatment with peace of mind. Help them ease their emotions, and they will be more cooperative with the various treatments in the future. (Participant 12).

Additionally, fighting cancer is a long process. Cancer patients may be affected by various factors, including disease progression, unsatisfactory treatment results, financial pressure, and family factors, which may affect their willingness and confidence regarding the continuation of treatment. Therefore, the participants stated that a large part of the so-called psychological care they provided to patients was intended to appease the patients, such as soothing and comforting them, and further to reduce patients’ anxiety and improve their confidence in the continuation of treatment.

Common psychological problems of cancer patients, as I just said, anxiety, fear…etc. Once diagnosed with cancer, patients may feel that it is a death sentence and have no confidence in receiving treatment. So in this situation, first of all, you have to relieve their anxiety and tell them that cancer is similar to a chronic disease, just like hypertension and diabetes. Under long-term drug control, the impact will not be great. Try not to let the patient think too much about it, and just do what they have to do for treatment. (Participant 15).

Some patients who have just been diagnosed with cancer may have psychological worries. When they come, they ask me if the disease is incurable, or if the disease is in an advanced stage, they may think about giving up treatment. Therefore, it may be important to have a conversation with the patient when they first come to the hospital. (Participant 14).

We encouraged them to continue to receive treatment, to cooperate with the doctor's treatment, and tell them that there was still hope for a cure. Tell them to have confidence. Just tell them more about other previous successful cases, so that they can increase their confidence and not be so overly depressed. (Participant 4).

#### The importance of trust in the relationship

According to the participants’ experiences, in order to successfully sooth and comfort patients, it is very important to first establish a trusting therapeutic interpersonal relationship with the patients. The trust relationship affects whether the patient is willing to reveal his or her own psychological troubles to the nurse and accept the nurse's advice, and then determines whether the patient's mood will be more stable after receiving comfort from the nurse and the patient thus more willing to receive treatment.

I think first of all they should be able to trust me, regardless of any patient. When you communicate with them, they must first trust you, and then they will be willing to open up and talk to you about their problems. If they don't trust you, they'll put you off, which is nothing but a waste of time, and I don't see any benefit to that. (Participant 19).

According to the participants’ experiences, building a trusting relationship requires spending time interacting with the patient, showing empathy in the process and, when necessary, demonstrating cancer-related expertise and skills, all of which can contribute to a trusting relationship with the patient.

It should be done step by step, and the natural way of communication is better… It is very important to gradually get them to trust you from unfamiliarity to familiarity. (Participant 2).

First of all, you must have a wealth of professional knowledge and skills worthy of their trust. The second is that you should chat more with the patient, and truly consider them from their perspectives. Only if you treat them sincerely can they gain trust in you. I think sincerity is also very important. (Participant 17).

I usually use my professional knowledge to convince the patient, and then I will gain the patient’s trust. (Participant 11)

#### The help of peer support

To comfort the patients, some participants would share the successful treatment experiences of other patients or introduce cancer patients to one another. Cancer patients share similar health problems and experiences in seeking medical treatment. The participants believed that the patient–patient interactions and sharing of experiences could achieve the effect of emotional catharsis for the patients and make them more confident in the treatment.

You can go to a patient who is taking the same drug, ask them to share their feelings about taking this drug, and let them communicate. Sometimes a patient may be more convincing than our medical staff. (Participant 6).

The patients will communicate with and encourage each other. In fact, they can be helped by mutual encouragement among patients. Especially for some patients who have just been diagnosed with cancer or have just started chemotherapy, this method is very useful for them. (Participant 10).

### Theme 3: A lack of psychology knowledge and communication skills

#### Not my expertise

Although all the participants had worked in the oncology ward for many years and considered psychological care to be part of their work, surprisingly, none of them had received any formal education or training on how to take care of the psychological needs of cancer patients. As a result, they generally expressed that they lacked relevant knowledge and skills. When they encountered emotional distress in the process of caring for cancer patients, they lacked confidence in their interactions with the patients. Some participants were not sure that they could help patients, even if they wanted to do so.

Because I am not an expert in psychology, I am just a nurse in the oncology department, and my knowledge of patients' emotional distress is quite limited. (Participant 11)

In terms of caring for patients, the more difficult part is that we lack some knowledge in psychological nursing, unlike others who have professional psychological counseling skills. (Participant 3).

After enlightening them, if they continue to immerse themselves in their own sadness, I will feel a little distressed, and I don't know how to comfort them, or what is the correct way to do it. (Participant 4).

#### A lack of communication skills

The participants generally believed communication skills to be very important when helping patients deal with emotional distress. However, some participants said that they lacked communication skills. In addition, some participants felt that there was a need to receive communication-related on-the-job education and training.

Sometimes I really don't know how to face patients, or what kind of psychological care will help them more. Because there are many times when I feel that I have said what I should say, but I have run out of words, and I feel that I have not achieved such a good effect, and I may be a little lacking in communication skills. (Participant 4).

I think some of the things I have to learn, such as communication skills or the psychological problems of cancer patients, and a method of communication with patients, because these things may be more helpful to me in clinical nursing practice. (Participant 7).

### Theme 4: Negative impacts of a lack of time

#### It takes time

For participants, dealing with the patients' emotional problems is not a simple matter, but a complicated and time-consuming task. It takes time to observe whether a patient is emotionally disturbed, spend time interacting with them, and establish a trusting relationship. Additionally, the way the nurses communicate with the patients will vary depending on the patients' personality traits or preferred communication methods. Therefore, it also takes time to understand patients’ personalities. Furthermore, for the participants, it may take a great deal of time to deal with the patients' emotional problems

First of all, you have to spend time observing patients, and learn to listen to what others say. As a good listener, I think it is also very important. Know how to listen, and know how to grasp some key words that patients say. From some key words spoken by the patient, I was keenly aware that her emotional state was not right. (Participant 2).

I think it's more about needing time to get familiar with each other. Especially, our nurses need time to understand the patients' personalities and then find a suitable way to communicate with them. Some people, you need to follow their words. For some people, you may need to interrupt them in time, correct them in time, explain to them what is wrong and what is right, and they can correct their thoughts in time. But there are also some people who are extreme. You have to follow "What she says is what she says.” (Participant 18).

In addition, the patient's emotional problems require the nursing staff to spend time and continuously intervene many times to deal with them. No single counseling interaction can achieve a great effect.

I think that after talking with a patient, there will be some effect, but the effect may not last for a long time. Maybe when you come to work the next day, you will find that the patient is still a little upset…so I think, a brief chat with the patient cannot fundamentally solve the patient's psychological problems… Sometimes they end up with a course of treatment, and the next time they come, they will still be depressed. (Participant 1).

#### Limited time available

Nurses have limited chances and time to interact with patients. The first reason is that the patient’s hospital stay is short and the schedule is full of treatments. The time available for interaction and interviews is limited.

Because the patients' hospitalization times are relatively short, about four or five days before and after, the time for communicating with them is very short. All I can say is that I made them a little happier. (Participant 19).

Some patients may be discharged just two or three days after being admitted to the hospital, and sometimes they are discharged without engaging in much communication with us. (Participant 2).

The second reason is that the nurses themselves are very busy with their work. The time and energy available for a nurse to pay attention to detect and deal with patients’ emotional distress is very tight. In this situation, the psychological problems of some cancer patients are likely to be ignored by nursing staff.

In fact, when it comes to actual psychological care for patients, I think it is still relatively lacking. After all, there are too few clinical nurses and too many patients. If we do this one by one, relative healing cannot be completed. The purpose of the patient’s visit is to see a doctor, and what the patient wants is to come for chemotherapy and to cure the disease, and to go home as soon as possible. Therefore, our focus is mostly on the chemotherapy. (Participant 19).

Psychological care is slightly less. I think that because I am too busy at work, I don't have so much time to talk to patients. Therefore, we can only pay additional attention to patients who are particularly depressed. (Participant 14).

### Theme 5: Managing emotional labor

#### Digesting the emotions of an angry patient

Emotional labor refers to the effort involved in managing feelings when the work role specifies that particular emotions should be displayed and others should be hidden. Managing emotional labor is something that nurses in cancer wards encounter in their daily work. Many participants mentioned that, in the process of caring for cancer patients, their emotions would be affected by the patients, but in order to appear professional, they could not show any emotions. As one participant stated:

I think nurses in our oncology department must have a strong psychological adjustment function. If you want to ease the emotions of patients, you must first control your emotions and not bring your emotions to patients. (Participant 3).

Additionally, most participants had similar experiences of interacting with angry patients or family members. They felt that they needed to regulate their emotions, which most likely had to do with the patients' anger. At work, they would encounter patients who were emotionally unstable and vented their anger on the nurses. These experiences affected their emotions and work attitudes, and the effects could even be lasting.

In the past, I often encountered people who swore and swore. If we encounter such patients or family members, we will complain to our colleagues, and we will try to limit our contact with them as much as possible…. If I encounter such an irritable patient at work, I feel that my mood will be affected a little bit, and I will not be interested in it, and I will not be as active and enthusiastic in taking care of other patients. (Participant 1).

#### Compassion and powerlessness

Additionally, there are many problems that cancer patients may face and worry about. In addition to disease progression, repeated hospitalization, and pain caused by treatment, they may also include psychological, family, and economic problems. While they are caring for and helping their patients, some nurses may be affected emotionally by the patients' situations, such as feeling powerless, feeling that their abilities are limited, and feeling that there is not much that can help the patient. Many participants said they also had to deal with the emotions that come with caring for patients.

I remember that he was very anxious when he came that time. He was worried about money, and there were still children at home. Later, his wife didn't come, and he was alone. I feel very sad… I don't think I have any good ways to help such a patient, because I can't help much financially. (Participant 7).

She felt a lot of pressure, so she chatted with me, and started crying after chatting, and I started crying when she cried, and finally the chat was a little bit unbearable. From then on, I knew that we really need to empathize with the patient, but we really can't empathize too deeply, because it will affect the quality of my care for her. My emotions were affected, and it took me a long time to move on from that experience. (Participant 19).

### Theme 6: Reflecting on the experiences

#### Accomplishment in doing good things

Reflecting on the experience of caring for cancer patients, most of the participants had positive attitudes towards individuals having the opportunity to assist cancer patients with reducing emotional distress during treatment.

I am also very happy to help them solve problems, because patients who have received chemotherapy for a long time will feel more or less in a bad mood after a long time. I am also very happy that I can help them feel a little better. (Participant 5).

I may have just said a few words, but for him, it made me feel proud that those words actually solved his problem. (Participant 10)

### Cherish personal and family health

Some participants sometimes reflected on their personal or family health or life attitudes and other values because of the patients' experiences and situations. For example, they cared more about or cherished their own and their families’ health.

Because I have worked in the oncology department for a long time, I will also become a little scared. Many times, I am also afraid that I will have physical problems someday, and I may reflect on such a thing. (Participant 4).

Taking care of cancer patients has had a great impact on my life. For example, it has had a greater impact on my expectations for my children. It doesn't matter if my children are not smart or have poor grades. I just hope that they will never get sick. (Participant 3).

After working in the oncology department for several years, I also worried about whether my family members would have cancer. I took them for regular physical examinations, and they also had gastrointestinal endoscopy, etc. For some early polyps, I recommend early intervention and early resection. (Participant 2).

## Discussion

The findings of our study add depth and richness to the meagre literature on how Chinese oncology nurses perceive their experiences of caring for cancer patients with emotional distress. These experiences were captured by six themes: (1) dictating the abnormality of emotion, (2) soothing and comforting patients, (3) a lack of psychology knowledge and communication skills, (4) negative impacts of a lack of time, (5) managing emotional labor and (6) reflecting on the experiences. Some critical issues around our themes and subthemes are worthy of further discussion.

### Dictating the abnormality of emotion

The results of this study suggest that, although emotional distress has been advocated for decades as a sixth vital sign in cancer patients [7], and screening for mental health distress is so important that it has been recommended as a standard of care [15], nurses are perceived as key in providing emotional support and quality care throughout the cancer treatment course [25]. But in some hospitals of China, this has not been implemented. Although the nurses in this study emphasized that it is part of the care, it seems that they did not regularly assess the patient's psychological state in actual practice, in the experiences of the individual cases. The oncology nurses in this study tended to, in the process of carrying out the daily care of the patient, incidentally observe whether the patient had signs of emotional distress, and then they would intervene to deal with abnormalities in emotion. From a certain point of view, it seems reasonable to do so, since observation is an effective strategy for understanding a patient's emotional state. It has been argued that patients do not necessarily respond proactively to their emotional distress, especially when they do not perceive a need. They might be unwilling to bother the healthcare professionals, whom they perceive as too busy, or they may perceive that disclosure may be no practical use or fear negative impacts [26]. Previous studies have also shown that the influence of Chinese culture can inhibit patients from disclosing their needs. Patients feel embarrassed about bothering the nurses, so they express their physical pain only when it has become intolerable [27]. Therefore, nurses need to be able to recognize both verbal and non-verbal cues [28].

### A lack of psychology knowledge and communication skills

However, the results of this study show that a more worrying phenomenon is that these clinical nurses may, for reasons such as lack of adequate education about cancer patients' mental health issues, feel that paying attention to the emotional states of patients is not their concern. A lack of relevant knowledge and skills, and a lack of communication skills, leads to a lack of confidence in providing patient care. Our finding is in line with that of a previous study, which revealed that oncology nurses expressed concern about their abilities to detect distress in patients with cancer. In that study, many of the oncology nurses reported that they felt they did not have the knowledge to perform psychosocial evaluations on patients [15].

### Soothing and comforting patients

Without systematic mental health-related education, clinical nurses learn from experience and help patients with emotional distress in ways that they think are good for the patients. Some are good strategies, such as establishing therapeutic interpersonal relationships with the patients. In the patient–nurse relationship, the nurse can choose to become involved in the patient’s cancer experience when the patient allows the nurse to share in the experience [12]. A sense of partnership has been observed to develop between nurses and patients as they built a relationship, providing a basis for the development of comfort, confidence, and trust [27]. Additionally, the nurses in this study also utilized the power of peers to help their patients. Bovbjerg et al. [29] argued that receiving peer support can address patients’ informational needs and empower them. Peer support also appears useful for enhancing psychosocial outcomes such as patients’ engagement with social support, reduction of distress and social isolation, and increased feelings of optimism and hope [29].

But sometimes, some methods and concepts of nursing staff are not necessarily in line with current scientific recommendations. For example, some nurses tended not to take the initiative to talk about the patient's condition, or to understand the patient's emotional state, so as to avoid stimulating the patient. Additionally, some nurses in this study perceived that patient should be emotionally stable and willing to cooperate with the treatment, regarded as the best interaction scenario. They viewed the patients' emotions as a potential barrier to performing therapeutic activities. This attitude also needs to be adjusted. It has been argued that currently, many nurses are educated under a biomedical model, with the result that the care that they provide often focuses only on a patient’s physical needs [28]. Our findings emphasize the need to allocate appropriate resources to help nurses identify and address the mental health concerns of patients. Nurses can receive basic training in systematically identifying mental health distress and, perhaps more importantly, providing initial responses to alleviate the emotional suffering of patients [15].

### Negative impacts of a lack of time

The heavy workload of the nurses is evident in the shortage of nurses and the time constraints they face as they perform many nursing routines and documentation procedures [27]. Additionally, because of the heavy workload, there is not enough time to interact with patients. As a result, they are unable to detect patients' emotional distress in a timely manner, unable to build relationships with their patients or understand their patients' problems in depth, and unable to actively assist patients with other problems, which seriously affects the quality of care. Similar findings were reported in previous studies, Burzotta & Noble’s study revealed that nurses’ perception of lack of time is a major barrier to psychological support provision within a therapeutic relationship. Consequently, supportive communication is likely to be neglected during a busy shift [15]. Additionally, nurses also reported being so overburdened with practical care responsibilities that they rarely had time to get to know patients and talk to them about their emotional well-being [15]. Furthermore, the oncology nurses in this study usually sought to finish the work at hand as soon as possible. Due to time constraints, formal or active evaluation of patients' emotional states was not their priority. Several studies have shown that nurses tend to focus more on the physical than on the psychosocial needs of cancer patients, and that the latter is often not considered part of the routine practice of nursing [28]. Our finding is similar to the findings by Chan et al., who argued that oncology settings are time-constrained, emotionally charged environments for nurses, and that providing psychosocial care for patients is a secondary concern [28]. Nurses involved in cancer care are facing ever greater demands and must deliver care more quickly for economic reasons and because of a worldwide shortage of nurses [28]. However, in the short term, overburdened nursing staff cannot effectively pay attention to the emotional distress of cancer patients, and this problem may be difficult to solve. It is therefore suggested that a different approach to facilitating a time-efficient assessment of emotional distress should include the use of reliable and valid screening measures for prevalent mental health disorders, such as anxiety and depression [15].

### Managing emotional labor and reflecting on the experiences

Caring for a cancer patient provides an opportunity for the nursing staff to reflect. On the one hand, they help patients to realize the value of the nursing profession. On the other hand, from witnessing the effects of the illness on the quality of life, work, and family relationships of the patients, they cherish their own and their families’ health and quality of life more. However, it is also worth mentioning that encounters with ill-tempered, angry and unreasonable patients in the clinic and empathizing with the patients' situations can have lasting effects and cause a psychological burden on the nurses. It is necessary for nurses to learn about and reflect upon the different forms of emotional labor. It is suggested that leadership and support are needed to deal with the nurses’ perception that their communication training has been ineffective and their ability to manage strong emotions is deficient. Communication skills, honed by making continuous opportunities to communicate available, as well as an understanding of emotional labor, need to be integrated with mindfulness in the nurses’ care of themselves and their patients [28].

## Conclusion

This study used a qualitative research method to understand the experiences of Chinese clinical nurses in caring for patients' psychological problems in oncology wards. The results of the study show that, although nurses agree on the importance of psychological care for cancer patients, they are limited by their lack of knowledge, skills and time, resulting in limited care and a lack of self-confidence in what they can provide. The research results also show that cancer nurses may also be emotionally affected during the process of caring for patients, and there is a psychological burden of emotional labor. In view of this, hospital directors should pay more attention to related phenomena, give due diligence to the importance of the psychological problems of cancer patients, reflect on the key factors affecting the quality of nursing care, and formulate corresponding effective policies for improvement. For example, pre-employment education and training and on-the-job education should be arranged to help nurses in cancer wards acquire mental health-related knowledge and skills, learn to effectively detect patients' psychological problems and distress during busy work schedules, and provide initial response to alleviate the emotional suffering of patients to meet the needs of the patients. In addition, administrative supervisors should also consider the personal emotional states of nursing staff and provide psychological support and emotional counseling as necessary.

## Data Availability

The results generated during the study are not publicly available due to ethical considerations but are available from the corresponding author on reasonable request.

## Abbreviations

CASP: Critical Appraisal Skills Programme
HCPs: Healthcare professionals
NCCN: The National Comprehensive Cancer Network
QOL: Quality of life
WHO: World Health Organization
